# Clinical relevance of systematic human papillomavirus (HPV) diagnosis in oral squamous cell carcinoma

**DOI:** 10.1186/1750-9378-7-13

**Published:** 2012-05-30

**Authors:** Chloé Bertolus, Patrick Goudot, Antoine Gessain, Nicolas Berthet

**Affiliations:** 1 Pitié-Salpêtrière Hospital, Department of Maxillofacial surgery, 47-83 boulevard de l’ Hôpital, Paris 75013, France; 2 Institut Pasteur, Epidemiology and Physiopathology of Oncogenic Viruses Unit, 28 rue du Docteur Roux, Paris 75015, France; 3 CNRS URA 3015, 28 rue du Docteur Roux, Paris, 75015, France

## Abstract

Head & Neck Cancer (HNC) is one of the most common malignancies worldwide and among oral neoplasias about 90-92% are squamous cell carcinomas (OSCC). Alcohol and tobacco consumption have been recognized as the main risk factors for development of OSCC. However, 10 to 20% of patients suffering from OSCC have no history of use of these substances. Clinico-pathological evidence suggests that we are dealing with virally-induced cancers, and that HPV should not be a relevant candidate. A systematic search of HPV in OSCC has no real relevance in current clinical practice even although it is still relevant in organized research protocols. Further studies are ongoing, with the aim of identifying other infectious agents, including viruses, in OSCC.

## View point

The search for an infectious cause of human cancers remains a matter of great interest in medicine. The discovery of a viral agent in a given tumor may have crucial implications not only for diagnosis and better understanding of the tumor’s physiopathology, but also on its prevention and/or treatment. This has been well illustrated in the link between cervical carcinoma and some high-risk types of human papillomavirus (HPV), and for liver cancer and hepatitis B/C infection. However, it is probable that other cancers have infectious origins that remain to be identified [1].

Head & Neck Cancer (HNC) is one of the most common malignancies worldwide, and is a high priority for public health authorities. Among oral neoplasias, about 90-92% are squamous cell carcinomas (OSCC), developing from the mucosa, with the remaining 8-10% being predominantly made up of lymphomas, sarcomas, and salivary gland tumors. For many years, alcohol and tobacco consumption have been recognized as the main risk factors for the development of OSCC. However, 10 to 20% of patients suffering from OSCC have no history of use of these substances. In this comment, we ask if, based on current available data, it is clinically relevant to carry out systematic HPV searches in cases of OSCC. The debate will be opened on the need to search for other infectious agents in OSCC.

Different viruses have been looked for in HNCs. Most of the studies, probably because of a certain analogy and similarity to the situation found in cervical carcinoma, have focused mainly on papillomaviruses, especially the high risk ones. Human papillomavirus has been strongly associated with oropharyngeal squamous cell carcinoma (OPSCC). However, the prevalence of HPV infection in OSCC is quite heterogeneous, varying from 17% to 85% [2,3]. Anatomically, the oral cavity includes the inner mucosal lips, the mucosal cheeks and vestibule, the mobile part of the tongue, the gums, the floor of the mouth, the intermaxillary region, and the hard and soft palate, but excludes the oropharynx (*i.e.*, the tonsils, the base of the tongue, and the oropharyngeal walls). Strictly speaking*,* the base of the tongue and the palatine tonsils are part of the anterior and lateral wall of the oropharynx [4], and belong to Waldeyer’s ring. Moreover, the histological characteristics of the mucosa lining the oral cavity and the oropharynx are quite different and constitute two distinct entities. However, in many studies concerning HPV prevalence in HNCs, the base of the tongue and the palatine tonsils have been considered part of the oral cavity, leading to misinterpretation of the results [5,6]. The implication of HPV in the development of OSCC, even if still a matter of debate, seems rather poor, and is probably overestimated because of the anatomical confusion described above [3]. As higher radiosensitivity has been reported for tonsil HPV-SCC [7], this confusion can have implications on patient care. These therapeutic results cannot be extrapolated to the oral cavity.

Besides alcohol and tobacco consumption, considered as the historical and still major etiological factors for OSCC, it is fair to hypothesize that infectious agents (and mainly viruses) could be associated and/or causally linked with a subset of OSCC. This hypothesis stems from the fact that OSCC in non-smoker non-drinkers is more frequently reported in an older population than alcohol-tobacco-related cancer [8]. This is similar to the situation with classic Kaposi sarcoma, non-HIV-associated primary effusion lymphoma (both associated with KSHV/HHV-8) and Merkel cell carcinoma being causally linked with the recently discovered new human polyomavirus. Among the possible infectious agents associated with OSCC, HPV is an unlikely candidate, as HPV-associated cancer is most frequently located in the oropharynx of young men [7].

Other clinico-pathological evidence suggests that we are indeed dealing with virally-induced cancers. Some histological studies have shown that HPV-related OPSCCs are non-keratinizing tumors, whereas non-HPV-related OPSCCs are keratinizing [9]. This correlation between viral infection and poor histological differentiation is not exclusive to HPV: it is also found in other virally-induced tumors. In nasopharyngeal carcinoma, for example, the keratinizing form could be less associated with Epstein-Barr virus [10]. In addition, an epidemiological study has demonstrated significant trends towards decreased differentiation in oral cavity tumors over the past 30 years [11]. Such an increase of undifferentiated cancers, which are thought to be “more virus-associated” [12], suggests that new or unknown infectious agents could be involved in OSCC in non-smoking, non-drinking patients. Altogether, these results suggest that, if not due to HPV involvement, the decreased differentiation of OSCC could be attributable to another viral agent. Some studies have been performed to look for other viruses as etiological causes, but without real success [13-15].

In conclusion, recent epidemiological data tend to indicate that the low diagnosis of HPV in OSCC is linked with an actual low prevalence, independent of technical and anatomical biases. Given our current knowledge, a systematic search for HPV in OSCC is therefore a waste of health service resources, and is only relevant in the research environment, such as in organized research protocols. However, further studies continue to search for other infectious agents, including viruses, in OSCC.

## Competing interests

The authors declared that they have no competing interest.

## Authors’ contributions

All authors participated in the preparation and writing of this comment.
